# Supplementary material for: The PARA-suite: PAR-CLIP specific sequence read simulation and processing
Source: PeerJ. 2016 Oct 27;4:e2619. doi: 10.7717/peerj.2619 (PMC5088580; doi:10.7717/peerj.2619)
Supplement: Supplemental Information 1 [file peerj-04-2619-s001.pdf]

## Supplementary Methods

For an overview of the performance of different read aligners and binding site detection algorithms on 10 simulated PAR-CLIP datasets, we calculated the precision, recall and accuracy for each. We considered all reads originating from simulated RBP-binding sites (with T–C conversions) as positives and those originating from other areas of the reference (simulated contaminations) as negatives. True positive and negative reads are those which are aligned correctly, whereas false positive and negative reads are those which are wrongly or not aligned (Table 1; Supplementary Table 3). We used BMix, PARalyzer and our hierarchical clustering to obtain the read clusters. Filtering of the clusters generated with the hierarchical clustering was performed as described in Section 2.2. A correctly reported binding site was considered a true positive, a falsely reported cluster (simulated contamination without elevated T–C conversions) as a false positive, an unreported binding site as a false negative and an unreported cluster (without T–C conversions) as a true negative (Supplementary Table 4). Unfortunately, BMix does not report false negative clusters (contaminations) and thus we were not able to calculate the recall nor the accuracy, but only the precision.

### Execution commands

*Quality and adapter trimming:*

```
cutadapt -e 0.05 -q 28 -m 18 -b $adapter -f fastq -o $output $input
```

*Alignment:*

```
bwa aln -n $n $hg38_reference $trimmed_input > $output.sai ($n in {1, 2, 0.01, 0.02, 0.04})
bwa samse $hg38_reference $output.sai $trimmed_input > $output.sam
bowtie -S -v 1 --best -m $n --strata $hg38_reference -q $trimmed_input $output.sam ($n in {1,
        2})
bowtie2 -x $hg38_reference -U $trimmed_input -S $output.sam
parasuite map --refine -q $trimmed_input -r $hg38_reference -t $hg38_transcriptome -o
        $output --parasuite-mm $X ($X in {1, 2, 3, -1})
STAR --genomeDir $hg38_reference --readFilesIn $trimmed_input --outFileNamePrefix $output
subjunc -u -n -i $hg38_reference -r $trimmed_input -o $output.sam
tophat -o $output $hg38_reference $trimmed_input
```

MosaikBuild -q $trimmed_input -out $mosaik_input -st illumina -ga hg38

MosaikAligner -ia $hg38_reference -in $mosaik_input -out $output -mm 3 -annse ./mosaik-2.2.3/network_files/2.1.78.se.ann -annpe ./mosaik-2.2.3/network_files/2.1.78.pe.ann -m unique -bw 5

*RBP binding site detection:*

PARalyzer config file:

BANDWIDTH=3

CONVERSION=T>C

MINIMUM_READ_COUNT_PER_CLUSTER=5

MINIMUM_READ_COUNT_FOR_KDE=3

MINIMUM_CLUSTER_SIZE=14

MINIMUM_CONVERSION_LOCATIONS_FOR_CLUSTER=1

MINIMUM_CONVERSION_COUNT_FOR_CLUSTER=1

MINIMUM_READ_COUNT_FOR_CLUSTER_INCLUSION=5

MINIMUM_READ_LENGTH=13

MAXIMUM_NUMBER_OF_NON_CONVERSION_MISMATCHES=0

MINIMUM_READ_COUNT_PER_GROUP=5

EXTEND_BY_READ

BMix config file:

COV_MIN=5

REFINE_COV=1

CONFIDENCE_PER=0.95

SEPARATE_STRANDS=1

PARA-suite clustering:

parasuite clust $alignment.bam $hg38_reference $output $dbsnp_142 5

*Annotation:*

annotatePeaks.pl $clusters.peak hg38 -norevopp -strand "+" > $clusters.annotated

## Supplementary Results

### Simulation of uridylate-rich and homopolymeric PAR-CLIP reads

To measure the accuracy of the PARA-suite aligner for special types of data (uridylate-rich sequences, which are common in PAR-CLIP and homopolymeric sequences), we generated subsets of our simulated data that contained either >35% T (uridylate-rich sequences) or homopolymeric sequences with stretches of five or more bases of a particular nucleotide.

For the uridylate-rich PAR-CLIP reads, we observed an increase of 1.37% for PARA-suite alignments and an increase of 2.35% in the accuracy for BWA PSSM alignments compared to our basic simulated data (Supplementary Table 5). The accuracy for the PARA-suite decreased by 1.53% but the accuracy was unchanged for BWA PSSM when the PARA-suite was applied to the homopolymeric PAR-CLIP reads (Supplementary Table 5).

### Application of the PARA-suite to HITS-CLIP data

Besides PAR-CLIP, other CLIP protocols are also used widely. Therefore, we chose a previously published Argonaute protein HITS-CLIP dataset generated from mouse brain samples (Chi, Zang et al. 2009) to assess the PARA-suite on a different type of CLIP data. To allow a comparison to previous results on the same dataset, we excluded all sequencing reads that were shorter than 25 bases after quality trimming using cutadapt. Next, we determined the error profile for the pooled replicates of the HITS-CLIP dataset using the respective PARA-suite tool to train its alignment pipeline. Here, we could already verify the high rate of deletions in contrast to insertions or single nucleotide substitutions compared to the mouse reference genome sequence GRCm38 (Chinwalla, Cook et al. 2002). Next, we applied the alignment pipeline to the pooled sequencing reads to align them against GRCm38 and against the transcript database of Ensembl genes Version 77 for the mouse genome assembly, and combined the results. Again, the transcriptomic mapping step revealed 79,658 additional aligned reads spanning exon–exon junctions out of 15,145,095 aligned reads in total (0.526 %). To achieve comparable results for RBP-bound transcribed regions in the mouse genome, we used PIPE-CLIP (Chen, Yun et al. 2014), which is a web-based program for cluster enrichment analysis of CLIP sequencing data. We compared our results with the number of cross-linked regions reported in the PIPE-CLIP publication analyzing the same

dataset. The filtering criteria were the same as those in the PIPE-CLIP publication with an enriched cluster length of ≥25 bases and exclusion of duplicated sequencing reads by mapping position. After filtering the entire list of cross-linked regions for those that were supported by deletions in the cross-linked sites, we found 1450 significantly enriched regions by applying false discovery rate (FDR) ≤0.01 filtering. This number was substantially larger than what was found by the initial PIPE-CLIP analysis based on read alignments using Novoalign (http://www.novocraft.com) with 1232 cross-linked regions that were supported by deletions, an increase of 17.69% identified regions in total.

We also applied FDR ≤0.001 filtering to compare our results with the first in-depth analysis of the same data (Zhang and Darnell 2011), which used a cross-linking-induced mutation sites (CIMS) analysis. We identified 984 cross-linked regions showing a reliable deletion, whereas the CIMS analysis applied to the read alignments performed by Novoalign identified only 886 cross-linked regions (Zhang and Darnell 2011).

## Supplementary Tables and Figures

**Supplementary Table S1:** Statistics of *FET* PAR-CLIP reads (Hoell, Larsson et al. 2011) before and after filtering for confident clusters.

| Dataset | Reads in clusters | Reads in confident clusters | % reads passing the filter |
|---|---|---|---|
| ***EWSR1*** | 1,375,517 | 700,936 | 50.96 |
| ***FUS*** | 1,249,406 | 923,904 | 73.95 |
| ***TAF15*** | 1,310,291 | 761,710 | 58.13 |

**Supplementary Table S2:** Average numbers for 10 simulated PAR-CLIP datasets.

| | |
|---|---|
| **Simulated reads** | 1,326,151 |
| **Mean read length** | 23 |
| **Clusters** | 85,691 |
| **T–C conversions** | 624,737 |
| **Sequencing errors** | 367,325 |
| **Indels** | 7324 |

**Supplementary Table S3:** Average performance of short read aligners on 10 simulated PAR-CLIP datasets sorted by accuracy. The runtime for BWA PARA was determined without error profile estimation, whereas the runtime for the entire PARA-suite pipeline includes error profile estimation, and alignment against genomic and transcriptomic reference sequences and both of these in combination. The results for "PARAsuite pipeline" refer to an execution where the parameter X was automatically evaluated (default). The results for "PARAsuite X1", "X2" and "X3" refer to executions with fixed values for X (i.e. X = 1, X = 2 and X = 3; see section "execution commands" for further information).

| **Aligner** | **Accuracy (in %)** | **Variance** | **Recall (in %)** | **Precision (in %)** | **Mapped overall** | **Mapped correctly** | **CPU time (in s)** | **Real time (in s)** | **Memory (in GB)** |
|---|---|---|---|---|---|---|---|---|---|
| **PARAsuite pipeline** | 73.14 | 1.37E-06 | 84.49 | 71.85 | 1,024,792 | 969,948 | 2287.3 | 396.8 | 6.27 |
| **PARAsuite X3 pipeline** | 72.61 | 1.26E-06 | 84.57 | 70.76 | 1,057,149 | 962,901 | 1365.9 | 307.7 | 6.21 |
| **PARAsuite X2 pipeline** | 71.63 | 1.31E-06 | 83.39 | 70.35 | 993,244 | 949,870 | 3786.6 | 539.2 | 6.33 |
| **PARAsuite** | 69.74 | 1.38E-06 | 82.16 | 68.24 | 975,672 | 924,802 | 1189.7 | 153.7 | 4.42 |
| **PARAsuite X3** | 68.57 | 1.46E-06 | 81.85 | 66.36 | 995,213 | 909,390 | 356.6 | 73.0 | 4.42 |
| **PARAsuite X2** | 68.26 | 1.33E-06 | 81.04 | 66.79 | 945,035 | 905,293 | 2405.1 | 265.1 | 4.42 |
| **BWA 002** | 68.17 | 1.38E-06 | 82.32 | 64.98 | 959,235 | 904,090 | 3621.9 | 359.2 | 4.42 |
| **BWA 004** | 68.17 | 1.37E-06 | 82.31 | 64.98 | 959,171 | 904,034 | 3981.5 | 390.7 | 4.42 |
| **BWA 2MM** | 68.17 | 1.37E-06 | 82.31 | 64.98 | 959,171 | 904,034 | 795.5 | 109.5 | 4.42 |
| **BWA 001** | 66.73 | 1.46E-06 | 80.61 | 64.26 | 958,919 | 884,964 | 797.2 | 109.5 | 4.42 |
| **Bowtie 2MM** | 63.38 | 1.10E-06 | 77.91 | 60.93 | 886,512 | 840,540 | 713.2 | 120.6 | 4.46 |
| **BWA PSSM** | 59.80 | 1.18E-06 | 74.04 | 58.72 | 818,895 | 793,007 | 232.4 | 25.4 | 2.26 |
| **TopHat** | 59.69 | 8.35E-07 | 76.10 | 55.35 | 844,902 | 791,549 | 592.9 | 282.9 | - |
| **BWA 1MM** | 59.29 | 8.68E-07 | 77.01 | 53.26 | 808,033 | 786,330 | 76.8 | 13.4 | 3.32 |
| **Bowtie2** | 56.22 | 1.11E-06 | 73.23 | 51.43 | 763,893 | 745,531 | 93.8 | 45.8 | 4.41 |
| **Bowtie 1mm** | 56.19 | 1.11E-06 | 73.20 | 51.42 | 763,631 | 745,227 | 1016.3 | 268.0 | 6.12 |
| **PARAsuite X1 pipeline** | 53.02 | 8.44E-07 | 68.55 | 51.20 | 716,838 | 703,161 | 54.0 | 10.8 | 2.26 |
| **PARAsuite X1** | 50.85 | 9.15E-07 | 66.52 | 49.08 | 685,788 | 674,399 | 75.0 | 43.7 | 4.41 |
| **STAR** | 50.74 | 9.10E-07 | 69.57 | 43.02 | 826,871 | 672,920 | 133.5 | 248.6 | 28.39 |
| **MOSAIK** | 44.88 | 2.18E-04 | 62.83 | 37.16 | 897,679 | 595,220 | 18,125.54 | 12,128.18 | 194.16 |
| **Subjunc** | 35.42 | 9.03E-07 | 50.61 | 26.09 | 597,400 | 469,751 | 24.3 | 64.2 | 6.65 |

**Supplementary TableS 4:** Binding sites detected by BMix, PARalyzer and the hierarchical clustering applied to read alignments of 10 simulated PAR-CLIP datasets. Recall and accuracy cannot be calculated for BMix because it does not provide a list of negative (discarded) clusters.

| Aligner | True positives | True negatives | False positives | False negatives | Precision (in %) |
|---|---|---|---|---|---|
| **BWA 2mm BMix** | 29,631 | 0 | 1456 | 0 | 95.32 |
| **BWA 2mm clustering** | 30,516 | 17,587 | 1795 | 5229 | 94.45 |
| **BWA 2mm paralyzer** | 29,255 | 12,184 | 5684 | 1575 | 83.73 |
| **BWA PSSM BMix** | 28,440 | 0 | 1470 | 0 | 95.09 |
| **BWA PSSM clustering** | 29,130 | 15,993 | 1837 | 2222 | 94.07 |
| **BWA PSSM paralyzer** | 28,396 | 11,172 | 5663 | 952 | 83.37 |
| **Bowtie 1mm BMix** | 26,824 | 0 | 969 | 0 | 96.51 |
| **Bowtie 1mm clustering** | 27,234 | 16,230 | 1137 | 3605 | 95.99 |
| **Bowtie 1mm paralyzer** | 27,464 | 11,252 | 5223 | 1299 | 84.02 |
| **Bowtie 2mm BMix** | 28,061 | 0 | 1375 | 0 | 95.33 |
| **Bowtie 2mm clustering** | 28,911 | 16,359 | 1691 | 4491 | 94.47 |
| **Bowtie 2mm paralyzer** | 27,979 | 11,218 | 5303 | 1280 | 84.07 |
| **Bowtie2 BMix** | 26,832 | 0 | 969 | 0 | 96.52 |
| **Bowtie2 clustering** | 27,231 | 16,239 | 1138 | 3611 | 95.99 |
| **Bowtie2 paralyzer** | 29,631 | 0 | 1456 | 0 | 84.03 |
| **PARA-suite BMix** | 31,918 | 0 | 1908 | 0 | 94.36 |
| **PARA-suite clustering** | 32,995 | 17,940 | 2394 | 4065 | 93.23 |
| **PARA-suite paralyzer** | 30,149 | 12,448 | 6329 | 2176 | 82.65 |

**Supplementary Table S5:** Alignment fractions of selected short read aligners applied to the PAR-CLIP results of the *FET* protein family. The PARA-suite aligner outperformed BWA 2MMs and BWA PSSM for all three datasets.

| Dataset | Reads after trimming | PARA-suite aligner | PARA-suite aligner fraction | BWA PSSM | BWA PSSM fraction | BWA 2MMs | BWA 2MMs fraction |
|---|---|---|---|---|---|---|---|
| ***EWSR1*** | 14,557,174 | 3,193,140 | 21.94% | 2,350,935 | 16.15% | 2,870,884 | 19.72% |
| ***FUS*** | 10,981,718 | 3,571,035 | 32.70% | 3,161,867 | 28.79% | 3,083,820 | 28.08% |
| ***TAF15*** | 10,611,969 | 2,457,585 | 23.16% | 1,605,642 | 15.13% | 2,326,287 | 21.92% |

**Supplementary Table S6:** Accuracy of the PARA-suite and BWA PSSM on uridylate-rich and homopolymeric simulated PAR-CLIP data.

| **Aligner** | **Accuracy** | |
|---|---|---|
| | **Uridylate-rich** | **Homopolymers** |
| **PARA-suite** | 71.11 | 68.21 |
| **BWA PSSM** | 62.15 | 59.80 |

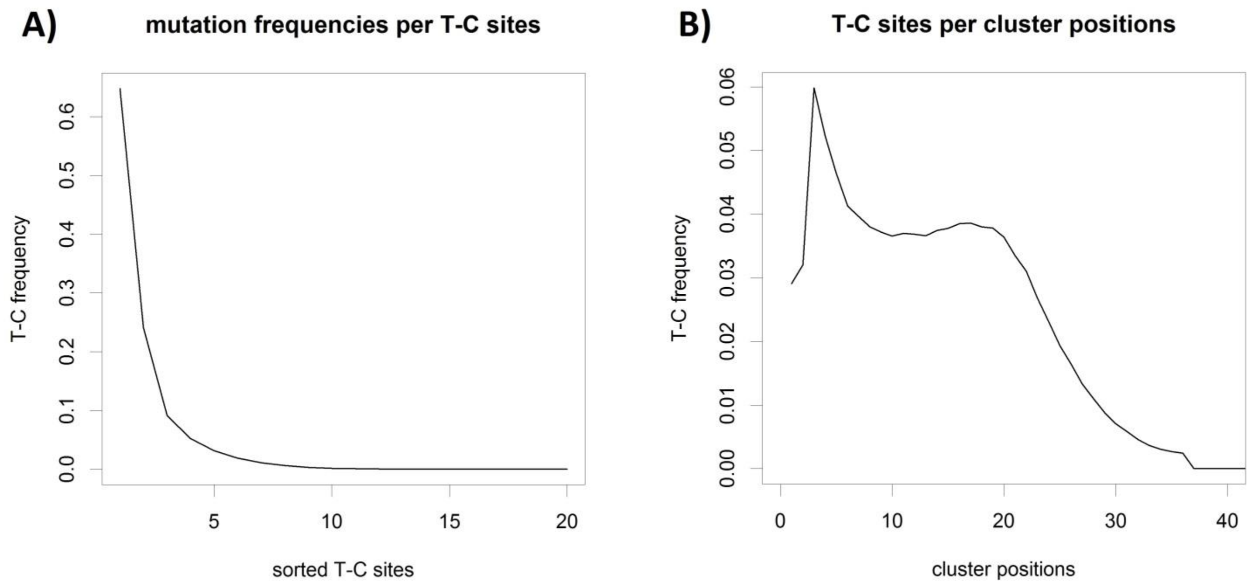


**Supplementary Figure S1:** (A) T–C conversion frequencies (α) in real PAR-CLIP data (summarized over all *FET* PAR-CLIPs (Hoell, Larsson et al. 2011)) and sorted by T–C sites within highly confident clusters. (B) Probabilities (β) for the preferred read positions of T–C conversion sites within confident clusters. This graph shows a peak at the beginning of the clusters where the majority of T–C conversions occurred.

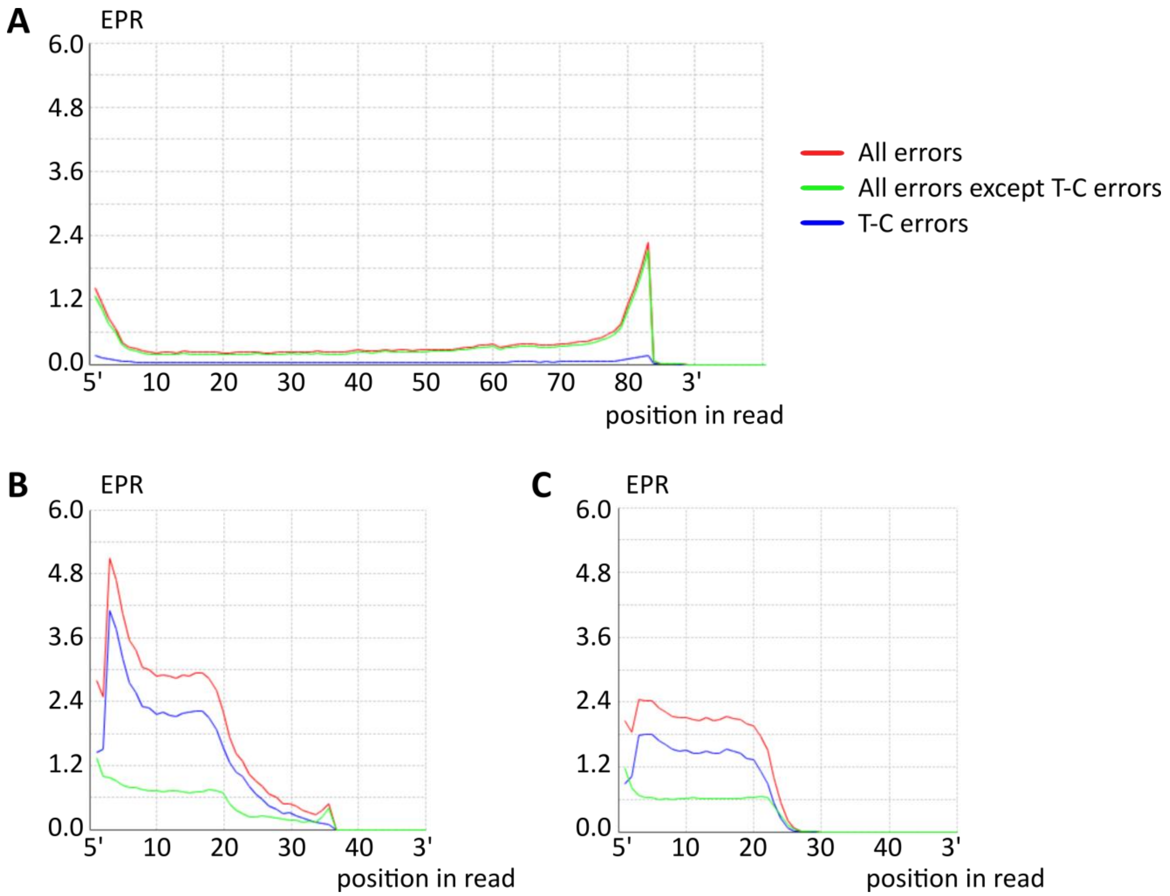


**Supplementary Figure S2:** Error profiles for (A) human reference RNA-Seq, (B) *FUS* PAR-CLIP and (C) simulated PAR-CLIP data (averaged over 10 simulated datasets) showing position-wise errors per reads × 100 (EPR). The RNA-Seq profile in (A) has higher sequencing error rates in the outermost bases and a very low average in the mid-range of the reads. The two PAR-CLIP error-profiles in (B) and (C) show a high increase in T–C errors between the read sequences and the reference sequence.

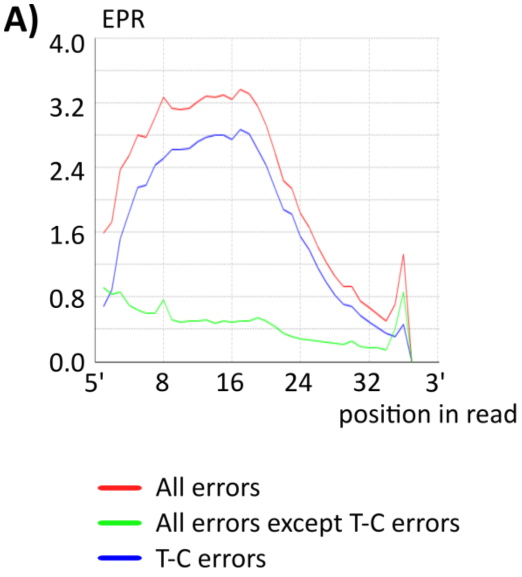


**Supplementary Figure S3:** Error profiles for (A) *HuR* (Mukherjee, Corcoran et al. 2011) and (B) *MOV10* (Sievers, Schlumpf et al. 2012). Both error profiles lack a peak in the error rate for the first bases but show nearly the same average T–C conversion frequencies as the *FET* PAR-CLIP dataset with 1.684 errors per reads × 100 (EPR) for *HuR* and 1.561 EPR for *MOV10* as compared to 1.477 EPR for, say, *FUS*.

A)

B)

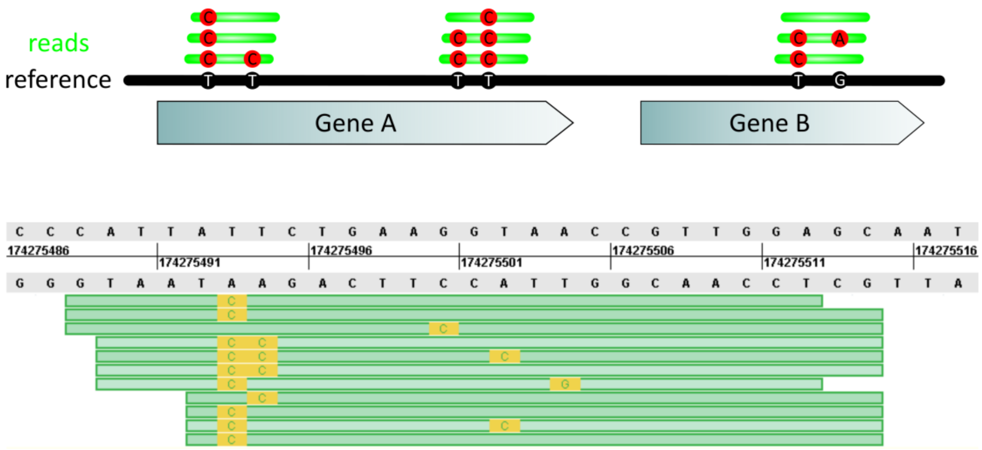


**Supplementary Figure S4:** (A) Schematic view of PAR-CLIP reads aligned against a reference sequence. All reads are stacked into three clusters covering only small parts of the respective genes. Furthermore, T–C conversion sites with high and low mutation frequencies as well as a G–A sequencing errors are shown. (B) Modified representation of a cluster of simulated PAR-CLIP sequencing reads, produced by GenomeView version 2350 ([http://genomeview.org/](http://genomeview.org/)). The cluster shows three T–C conversion sites, one of which has a very high amount of T–C conversions, and A–G and G–C sequencing errors.

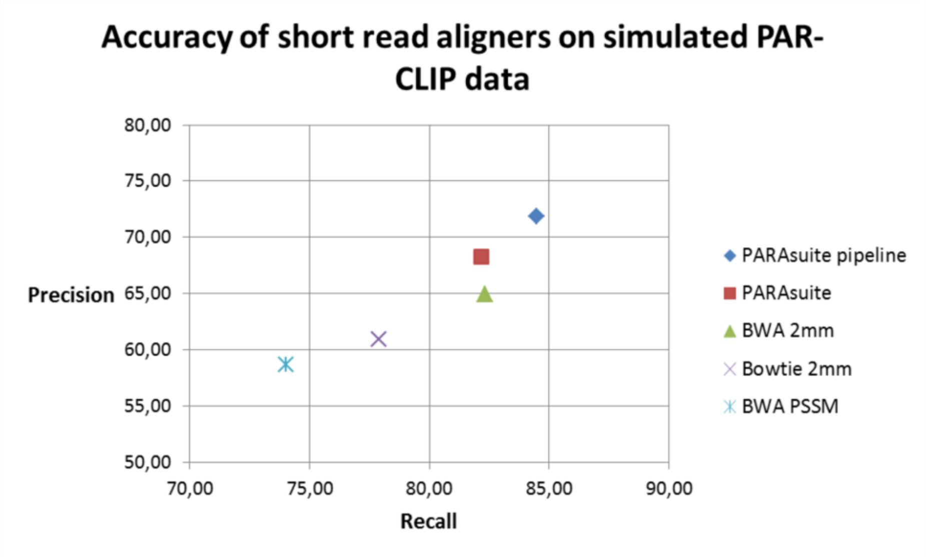


**Supplementary Figure S5:** Average accuracy of short read aligners on 10 simulated PAR-CLIP datasets. Bowtie and BWA were run allowing for two mismatches (Bowtie 2MMs and BWA 2MMs). The PARA-suite, including the transcriptome alignment (called the PARA-suite pipeline), outperformed all other aligners in recall and precision. The performance values obtained for additional aligners are listed in Supplementary Table 2.

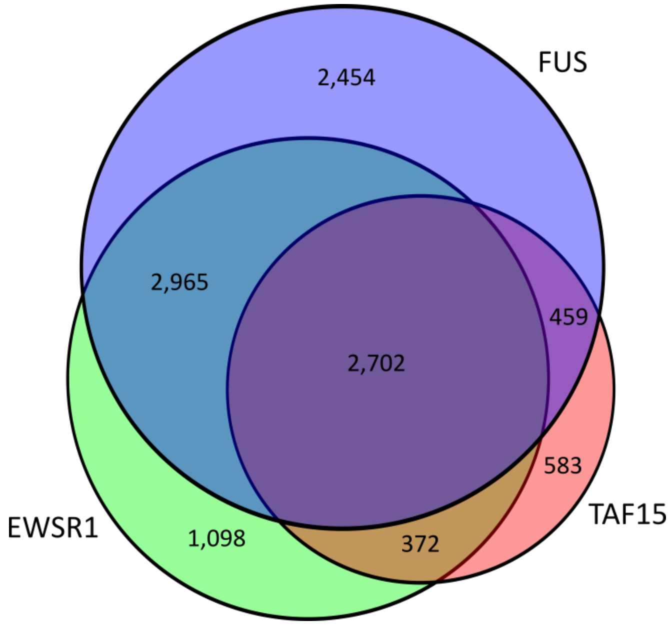


**Supplementary Figure S6:** Overlaps of genes targeted by the *FET* family identified by the cross-linked regions after cluster filtering. *P*-values for the Pairwise enrichments are as follows using Fisher's exact test: EWSR1–FUS enrichment = 2.1 (p-value < 0.000); FUS–TAF15 enrichment = 2.0 (p-value < 0.000); EWSR1–TAF15 enrichment = 2.4 (p-value < 0.000). The largest fraction of 2702 distinct genes is covered by all three datasets, which correlates with the results of the initial study.

## Supplementary References


Chen, B., Yun, J., Kim, M. S., Mendell, J. T. and Xie, Y. (2014). PIPE-CLIP: a comprehensive online tool for CLIP-seq data analysis. *Genome Biol.* **15**: R18.

Chi, S. W., Zang, J. B., Mele, A. and Darnell, R. B. (2009). Argonaute HITS-CLIP decodes microRNA–mRNA interaction maps. *Nature* **460**(7254): 479-486.

Chinwalla, A. T., Cook, L. L., Delehaunty, K. D., Fewell, G. A., Fulton, L. A., Fulton, R. S., Graves, T. A., Hillier, L. W., Mardis, E. R. and McPherson, J. D. (2002). Initial sequencing and comparative analysis of the mouse genome. *Nature* **420**(6915): 520-562.

Hoell, J. I., Larsson, E., Runge, S., Nusbaum, J. D., Duggimpudi, S., Farazi, T. A., Hafner, M., Borkhardt, A., Sander, C. and Tuschl, T. (2011). RNA targets of wild-type and mutant FET family proteins. *Nat. Struct. Mol. Biol.* **18**(12): 1428-1431.

Mukherjee, N., Corcoran, D. L., Nusbaum, J. D., Reid, D. W., Georgiev, S., Hafner, M., Ascano, M., Tuschl, T., Ohler, U. and Keene, J. D. (2011). Integrative regulatory mapping indicates that the RNA-binding protein HuR couples pre-mRNA processing and mRNA stability. *Mol. Cell* **43**(3): 327-339.

Sievers, C., Schlumpf, T., Sawarkar, R., Comoglio, F. and Paro, R. (2012). Mixture models and wavelet transforms reveal high confidence RNA-protein interaction sites in MOV10 PAR-CLIP data. *Nucleic Acids Res.* **40**(20): e160.

Zhang, C. and Darnell, R. B. (2011). Mapping in vivo protein-RNA interactions at single-nucleotide resolution from HITS-CLIP data. *Nat. Biotechnol.* **29**(7): 607-614.
